# Mapping generative AI in higher education teaching and learning: a scoping review

**DOI:** 10.1186/s41239-026-00630-9

**Published:** 2026-10-15

**Authors:** Michael Pin-Chuan Lin, Daniel H. Chang, Philip H. Winne, G. Michael Bowen, Jeeho Ryoo

**Affiliations:** 1 Faculty of Education, Mount Saint Vincent University, Halifax, Canada; 2 Faculty of Education, Simon Fraser University, Burnaby, Canada; 3 Faculty of Humanities and Social Sciences, Athabasca University, Athabasca, Canada; 4 Olsen College of Engineering and Science, Fairleigh Dickinson University, Vancouver, Canada

**Keywords:** Generative AI, Large language models, Higher education, Teaching and learning, Assessment, Stakeholder perceptions, Scoping review

## Abstract

Generative artificial intelligence (GenAI) has rapidly entered higher education, but empirical research on its roles in teaching and learning remains fragmented across instructional activities, outcomes, assessment approaches, and stakeholder perspectives. This scoping review followed PRISMA-ScR guidelines to map 394 empirical studies of GenAI use in higher education published from November 2022 through December 2025. The review examined how GenAI tools were integrated into authentic teaching and learning activities, what outcomes or skills were targeted, how studies assessed those outcomes, and how students and instructors perceived GenAI use. Across the reviewed studies, GenAI was most often used to support existing academic tasks, especially feedback, tutoring, and writing. The most frequently targeted outcomes included engagement, usability and acceptance, critical thinking, disciplinary knowledge, academic performance, writing quality, and AI literacy. Self-regulated learning, ethics, creativity, feedback quality, and equity received less attention. Assessment practices relied mainly on surveys, tests or rubrics, artifact analysis, and interviews, suggesting that much of the evidence base is shaped by short-term and readily implemented methods. Student and instructor perceptions were predominantly mixed, reflecting perceived benefits alongside concerns about pedagogy, integrity, workload, and governance. The review highlights the need for stronger alignment between learning constructs and assessment methods, more process-sensitive evidence, clearer reporting of governance practices, and greater attention to equity in GenAI-supported higher education.

**Supplementary Information:**

The online version contains supplementary material available at https://doi.org/10.1186/s41239-026-00630-9.

## Introduction

Artificial intelligence (AI) is increasingly reshaping education regarding how students learn, how instructors teach, and how institutions organize educational practice (Chang et al., 2023; Ouyang & Jiao, 2021). Over the past decade, AI in education (AIEd) has developed into a broad field of study encompassing AI applications such as prediction, personalization, feedback, learning analytics, and instructional support, alongside multiple conceptual and ethical questions these technologies present (Chiu et al., 2023; Crompton & Burke, 2023; Ouyang & Jiao, 2021; Zawacki-Richter et al., 2019; Zhai et al., 2021). More recently, GenAI, especially large language model-based tools such as ChatGPT, has accelerated shifts in higher education. Unlike many earlier AIEd systems, these tools are directly accessible by students and instructors, usable across disciplines, and readily integrated into everyday academic tasks.

In higher education, AI systems broadly have been used to evaluate academic progress, predict students at-risk (Embarak & Hawarna, 2024), offer personalized assistance and adaptive learning support (Chang et al., 2023; Yao & González-Vélez, 2025), and support instructional design and learning analytics (Crompton & Burke, 2023; Darvishi et al., 2022; Liang & Luo, 2024). Applications of GenAI support activities such as writing, coding, idea generation, feedback, language learning, and content comprehension, particularly in contexts where text- or code-based work is central (Guo et al., 2024). Most adoptions appear pragmatic. Rather than fundamentally restructuring curriculum, GenAI has mainly been used to augment existing learning activities by providing on-demand assistance, accelerating task completion, or expanding access to feedback and support (Zhai et al., 2021). These developments have intensified debate about what counts as meaningful learning when students use GenAI and how its educational value should be examined in higher education (Noroozi et al., 2024).

Although empirical research on GenAI in higher education is expanding rapidly, the literature remains fragmented in several key ways. First, studies vary widely in how GenAI is used in teaching and learning, outcomes or skills are targeted, and how outcomes are conceptualized. Prior work (e.g., Chiu et al., 2023; Yu et al., 2024) has examined learning gains, writing quality, critical thinking, engagement, efficiency, usability, and affective responses, often using an idiosyncratic framework connecting outcomes to specific uses of GenAI. As a result, it remains difficult to discern which types of educational activities receive the most empirical attention and which learning priorities are being foregrounded.

Second, how outcomes are assessed is often unclear. This matters because claims about the educational value of GenAI depend not only on which outcomes are examined, but also on how researchers operationalize and evaluate them. When studies rely primarily on self-report, immediate performance, or post-task perceptions, the kinds of conclusions that can be drawn differ from those supported by trace data, longitudinal artifact analysis, external ratings, or observation. Mapping assessment approaches is therefore crucial for understanding the current evidence base and the kinds of conclusions that evidence can validly support.

Third, stakeholder perspectives have become central to discussions of GenAI in higher education (Chiu et al., 2023; Ifenthaler et al., 2024). Students and instructors are positioned differently in relation to these tools and may interpret their usefulness, risks, and legitimacy differently. Existing studies have raised recurring concerns about academic integrity, responsible use, disclosure, and institutional accountability, while also pointing to potential benefits for efficiency, support, and learning (Chang et al., 2023; Gruenhagen et al., 2024; Yusuf et al., 2024). However, empirical work varies considerably regarding which perspectives are examined and how they are reported. This makes it difficult to compare students’ with instructors’ viewpoints, and to understand how governance and policy issues are being addressed in higher education contexts in practice.

Existing systematic reviews and scoping reviews offer valuable classifications of how AI applications have been used in K-12 (Ma et al., 2025) or teacher education (Sperling et al., 2024), how AI has been incorporated in education (Chiu et al., 2023; Yu et al., 2024) or how ethical considerations have been conceptualized in AI-mediated learning environments (Wiese et al., 2025). These reviews have made important contributions, but several limitations remain. Many aggregate AI technologies broadly instead of distinguishing GenAI from earlier AIEd systems; some focus on sectors other than higher education; and others emphasize conceptual, policy, or meta-level discussions more than actual empirical use in authentic teaching and learning settings. Given the speed with which GenAI has been adopted, there is a need for an updated mapping of recent empirical work that focuses specifically on higher education and simultaneously considers instructional use, targeted outcomes, assessment practices, and stakeholder perceptions.

In response, this scoping review maps recent empirical studies of GenAI use in higher education. Unlike prior reviews that synthesize AI or GenAI in education at broader levels (e.g., Bahroun et al., 2023; Crompton & Burke, 2023; Noroozi et al., 2024), this review focuses specifically on enacted, task-anchored GenAI use in authentic higher education teaching and learning contexts. It examines how GenAI is being incorporated into teaching and learning activities, what educational outcomes or skills are targeted, how assessment methods are used across studies, and how students and instructors perceive its use, including concerns related to ethics, governance, and policy. By doing so, this review clarifies current patterns, identifies recurring emphases, and reveals gaps that need filling to develop more theoretically grounded and methodologically robust research. Specifically, we address these research questions (RQs):

1. How have GenAI tools been incorporated into authentic teaching and learning activities in higher education, and what educational outcomes or skills have empirical studies targeted?
2. What methods have empirical studies used to evaluate the educational outcomes or skills associated with GenAI use in higher education?
3. How do empirical studies of enacted GenAI use in higher education report students’ and instructors’ perceptions concerning effectiveness, ethical issues, and governance or policy implications?

## Methods

This scoping review provides a comprehensive account of the scope, volume, and focus of the research in higher education. The methodology follows the checklist of the PRISMA extension for scoping reviews (PRISMA-ScR) (Peters et al., 2020; Tricco et al., 2018) and the methodological framework outlined in Arksey and O’Malley (2005). The review process encompassed five key stages: (1) identifying scoping review RQs, (2) identifying relevant studies, (3) selecting studies, (4) charting the data, and (5) summarizing and reporting results.

### Literature search

The literature search was conducted in January 2026 to capture research on LLM-based GenAI in higher education teaching and learning following ChatGPT’s public release in November 2022, which accelerated GenAI uptake in higher education. To maximize coverage in a rapidly evolving field and to capture early-online and conference-linked journal articles that are subsequently indexed, we searched EBSCOhost and Web of Science Core Collection. Together, these resources offer broad cross-disciplinary indexing and reduce reliance on any single database’s coverage. The following search terms were used with Boolean logic for text appearing in a title or abstract: (“generative AI” OR “generative artificial intelligence” OR “ChatGPT” OR “large language model*” OR “LLM*”) AND (“teaching” OR “learning” OR “instruction” OR “education”) AND (“higher education” OR “college” OR “university” OR “post-secondary” OR “postsecondary” OR “undergraduate”). Search limits were set to peer-reviewed journal articles, English language, and publication dates from November 2022 through December 2025. The full database-specific search strategies, limits, and exported record counts are provided in Appendix A.

### Inclusion and exclusion criteria

To maintain methodological rigor and conceptual alignment with task-anchored GenAI use in higher education, we applied prespecified inclusion and exclusion criteria at both screening and full-text stages of review.

Studies were included if they (a) reported original empirical research (qualitative, quantitative, or mixed methods); (b) were situated in a higher education context (college, university, or other post-secondary settings) involving learners and/or instructors; (c) examined the use of GenAI tools (e.g., ChatGPT and any other large language model–enabled system); and (d) documented use in higher education teaching and learning. Specifically, GenAI use had to be embedded in a course-related task or instructional activity, such as an assignment, assessment, feedback cycle, instructional intervention, or instructor-led class activity, and involve students and/or instructors using GenAI.

Studies were excluded if they (a) lacked direct evidence of GenAI use embedded in an education-related task or learning activity; (b) involved GenAI primarily as a research instrument to generate artifacts that were then evaluated, rated, or benchmarked (for example, output-quality evaluation, prompt-engineering comparisons, rubric-alignment checks) without evidence that the outputs were used within an authentic teaching/learning activity; (c) were not situated in higher education; (d) were non-empirical (for example, opinion pieces, editorials, conceptual papers) or review articles; or (e) did not provide sufficient methodological detail to verify that GenAI use occurred in an authentic educational context, in which case the record was conservatively coded as unclear and excluded at full-text.

### Study selection

Eligibility screening was conducted using the inclusion and exclusion criteria described above. Titles and abstracts of the records were screened, and records that appeared eligible or could not be confidently excluded were advanced to full-text review. Three researchers participated in the full-text eligibility assessment of 523 articles. Before synthesis, eligibility decisions were checked against the finalized inclusion and exclusion criteria. Ambiguous or borderline cases were discussed by the review team and resolved by consensus. Records that did not meet the finalized criteria were excluded before analysis.

**Fig. 1:**
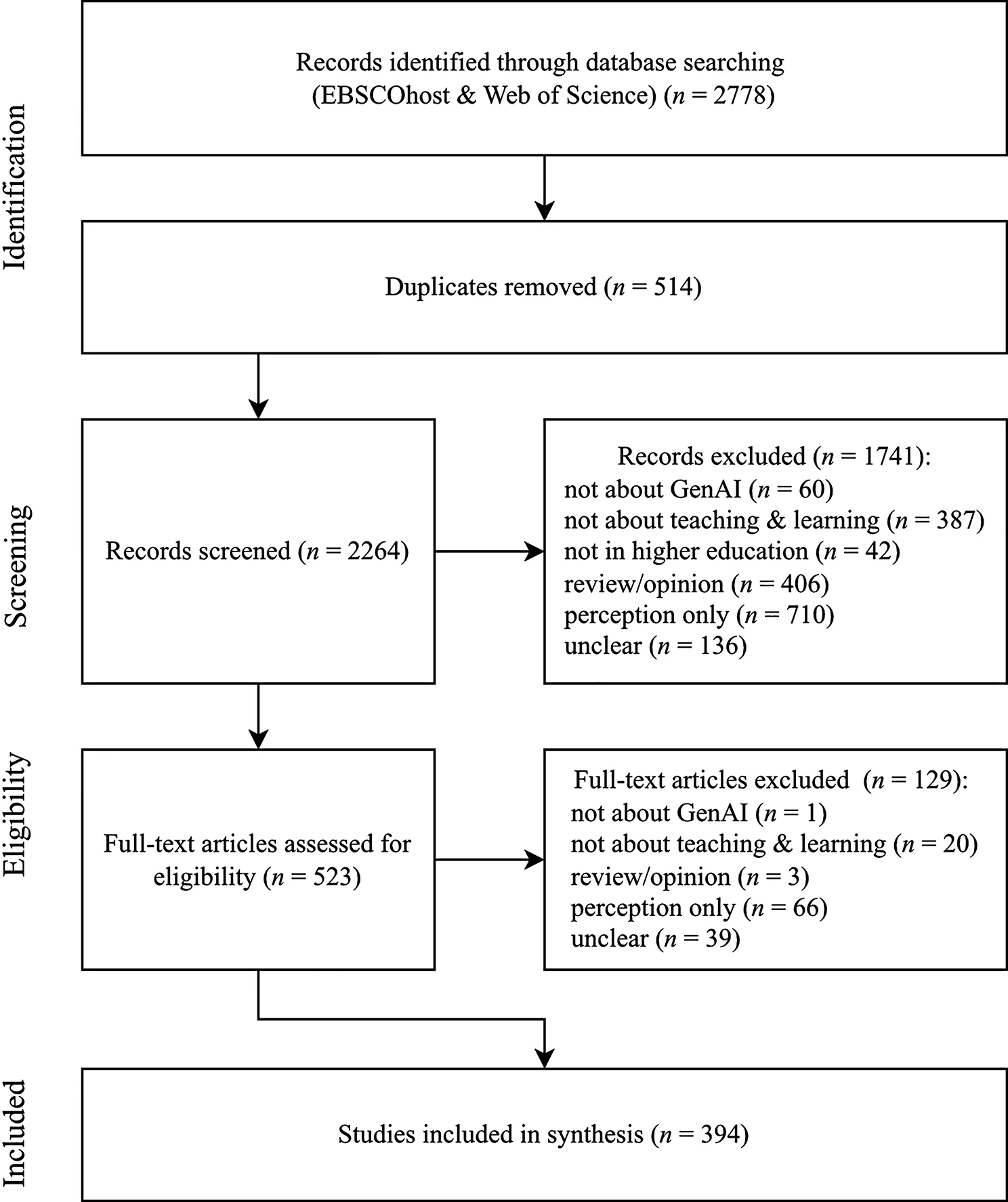
PRISMA diagram of article inclusion

Figure 1 summarizes the study identification and selection process using a PRISMA-style flow diagram. The database search returned 2,778 records (EBSCO = 976; Web of Science = 1,802). After removing 514 duplicates, 2,264 unique records remained for title and abstract screening. Screening excluded records that were not about GenAI (*n* = 60), not about teaching and learning (*n* = 387), not situated in higher education (*n* = 42), opinion or review articles (*n* = 406), perception-only studies with no enacted course use (*n* = 710), or unclear (*n* = 136), yielding 523 articles for full-text eligibility assessment. Full-text review resulted in 394 included empirical studies, with exclusions due to not about GenAI (*n* = 1), not about teaching and learning (*n* = 20), opinion/review (*n* = 3), perception-only studies with no enacted course use (*n* = 66), or unclear (*n* = 39).

### Coding and data analysis

Data charting was conducted using a master coding spreadsheet aligned with the RQs. Extracted fields captured study and sample characteristics, GenAI tool information, descriptive evidence for GenAI use-case coding, targeted outcomes, assessment methods, stakeholder perceptions, and ethics/policy indicators. Before full data charting, three researchers jointly coded a calibration set of 10 included studies to align coding interpretations, evidence rules, and decision rules for ambiguous cases. After calibration, the remaining included studies were divided among the three researchers for primary charting. Coders flagged ambiguous cases in the master spreadsheet and discussed them during virtual team check-ins. When needed, a second researcher reread the article before a final decision was entered. Final coding decisions were made by consensus. For reporting and synthesis, the analytic variables used in the descriptive mapping were compiled in Supplementary Table S1.

Coding focused on five sets of variables aligned with the aims of this review: (1) study profile and design, (2) GenAI use cases and targeted outcomes, (3) assessment methods, (4) stakeholder perceptions, and (5) ethics/policy and governance indicators. These variables were operationalized through predefined sub-categories and, where appropriate, refined through calibration coding, evidence-based coding rules, and consensus discussion. Table 1 summarizes the main analytic coding categories used for data charting and synthesis. The full operational codebook, including allowable values, coding rules, examples, and decision rules for ambiguous cases, is provided in Appendix B.

For RQ1, each study was assigned one single-label “use_case_category.” Coders first recorded how each article described the purpose of GenAI use and the enacted learning activity or task; these descriptive entries served as coding evidence rather than separate analytic outcomes. The final “use_case_category” reflected the GenAI-supported activity most central to the study aim, intervention, task design, enacted activity, or primary outcome. Initial categories were informed by prior AI/GenAI education scoping review frameworks (Joksimovic et al., 2023; Su & Yang, 2022; Xia et al., 2024), and final categories were refined against the included corpus through calibration coding, review-team discussion, and consensus adjudication. Final use-case categories are listed in Table 1 and operationalized in Appendix B.

Within the targeted outcomes code, achievement/task performance referred to demonstrated performance on assessed tasks, such as grades, scores, rubric-based outcomes, task accuracy, or task completion, whereas domain knowledge/conceptual understanding referred to subject-matter understanding or knowledge acquisition not necessarily measured through formal performance evaluation. Because studies could target more than one educational outcome or skill, targeted outcomes were coded as multi-label variables.

Assessment methods were also coded as multi-label variables because studies could use more than one method to evaluate targeted outcomes or skills. These codes captured the methods used to generate empirical evidence about GenAI-related learning, performance, perception, or process outcomes, including surveys, tests/quizzes/rubrics, artifact analysis, interviews/focus groups, analytics/logs, observation, expert ratings, and physiological or biometric measures.

For student and instructor perception, initial coding drew on sensitizing concepts from prior review literature (e.g., Chan, 2023; Kim et al., 2025; Xia et al., 2024), and candidate themes were refined inductively through calibration and check-in discussions in which researchers compared interpretations, consolidated overlapping labels, and agreed on final theme definitions. Perception direction was coded separately for student and instructor evidence as Positive, Negative, Mixed, or Not reported.

Within the ethics and policy category, we first coded three separate binary indicators: (a) whether ethics or policy issues were mentioned in relation to GenAI use, (b) whether the authors recommended a concrete policy, guideline, rule set, or governance action, and (c) whether the study reported instructor- or course-level rules governing GenAI use in the enacted learning context. These indicators were allowed to co-occur within the same study. For synthesis, we then created a derived single-label variable, “policy_presence”, to classify each study according to the highest level of governance explicitness reported. The hierarchy was applied as follows: if “AI_policy_recommend” = Yes, the study was classified as “institutional policy recommended”; otherwise, if “AI_policy_by_instructor” = Yes, it was classified as “instructor-level rules set”; otherwise, if “ethics_policy_issues_mentioned” = Yes, it was classified as “ethics/policy mentioned only”; otherwise, it was classified as “none mentioned.” This derived variable enabled each study to be counted once in the descriptive synthesis while preserving the underlying binary indicators in the coded dataset (Arksey & O’Malley, 2005; Levac et al., 2010; Tricco et al., 2018).

Analysis combined descriptive frequency counts with inductive content analysis to synthesize patterns within open-text fields and narrative findings (Mayring, 2015). For single-label variables, each study was counted once for the relevant variable. For multi-label variables, including targeted outcomes, assessment methods, and student/instructor perception themes, each category was treated as a binary present/absent indicator so that a study could contribute to more than one category when supported by article evidence. Assessment methods and targeted outcomes were coded as separate study-level multi-label variables; accordingly, the synthesis reports the distribution of assessment methods across studies rather than assigning specific methods to specific outcomes within multi-method studies. The derived “policy_presence” variable was generated after the three ethics/policy indicators were finalized and was used only as a single-label summary variable in the descriptive synthesis.

**Table 1:** Summarized coding scheme

Category	Code	Definition	Example
Basic Information	Author Year	Authoring information Year of publication	John & Chang 2024
Purpose and Domain	Use-case category	A single-label analytic variable capturing the primary enacted GenAI-supported teaching or learning activity in each study. Initial coding was informed by prior AI/GenAI education scoping reviews (Joksimovic et al., 2023; Su & Yang, 2022; Xia et al., 2024), and final categories were refined against the included corpus. Each study was assigned one primary use-case category based on the activity most central to the study aim, intervention, task design, or primary outcome. Final categories were: Feedback/tutoring; Writing support; Lesson planning; Language learning/translation; Programming/debugging; Assessment/grading; Idea generation; Research/literature support; General academic support; Simulation/role-play; Content creation/multimodal; Improve critical thinking; Reflection/SRL.	A study in which students used ChatGPT to receive formative comments on draft essays was coded as Feedback/tutoring.
Study Design	Research Design	Whether the methodology is qualitative, quantitative, or mixed methods.	Qualitative
	Participants	The group of individuals involved in the study.	Undergraduate students
	Targeted Skills/ Educational outcomes	Based on Weng et al. (2024) and Xia et al. (2024), this code identifies the skills or learning outcomes that studies aimed to develop or measure through GenAI applications in higher education. These outcomes represent the dependent variables used to evaluate the educational impact of Examples include critical thinking, achievement, domain knowledge, writing quality, AI literacy, and SRL/metacognition. GenAI tools.	Programming performance
	Assessment methods	The tools or methods used to evaluate the dependent variables or outcomes in the study.	Pretest and posttest
User Perception	Student perception	This code captures the dimensions of students’ perceptions toward GenAI in higher education (Kim et al., 2025; Xia et al., 2024). During coding, responses and findings were grouped into themes such as engagement/motivation, learning and skill development, reliability/usability, pedagogy/assessment, efficiency/workload, language support/writing quality, critical thinking, ethics/integrity, creativity/idea generation, dependence/overreliance, social and career impacts, equity/inclusion, privacy/data security, and policy/disclosure/governance.	Survey examining students’ perceptions of ChatGPT’s usefulness and convenience for coursework.
	Instructor perception	This code captures the dimensions of instructors’ perceptions regarding the pedagogical, practical, and ethical implications of GenAI (Xia et al., 2024; Chan, 2023). During coding, findings were grouped into themes such as efficiency/workload, pedagogy/assessment, ethics/integrity, professional learning/AI literacy, learning and skill development, reliability/usability, affect/technostress, engagement/motivation, equity/inclusion, critical thinking, policy/disclosure/governance, dependence/overreliance, creativity/idea generation, privacy/data security, and social and career impacts.	Interview responses on ChatGPT’s impact on assessment design and instructional practices.
Ethics and Policy	Ethics/policy issues mentioned	Whether the study addressed ethical considerations, policy implications, or guidelines related to AI use in educational contexts.	Students raised academic integrity and privacy concerns when using ChatGPT for coursework.
	AI policy recommend	Whether the authors explicitly recommend a concrete policy, guideline, rule set, or governance action for GenAI use (institutional, departmental, program, or course level) in the authors’ own voice (typically Discussion/Conclusion/Implications).	The authors recommend that universities establish disclosure guidelines for GenAI-assisted work.
	AI policy by instructor	Whether the study reports that the instructor/course set explicit rules or parameters governing GenAI use for the learning task(s) (e.g., permitted/prohibited uses, disclosure requirement, boundaries for prompting, or use of a structured framework to define allowable use).	Students were required to disclose prompts and could use GenAI for brainstorming but not for final drafting.
	Policy presence (derived)	A derived single-label variable summarizing the highest level of ethics/policy or governance explicitness reported in each study. The variable was constructed from three independent binary indicators: “ethics_policy_issues_mentioned”, “AI_policy_recommend”, and “AI_policy_by_instructor.” Because these indicators could co-occur, a hierarchy was applied so that each study was counted once in the descriptive synthesis: (3) institutional policy recommended; (2) instructor-level rules set; (1) ethics/policy mentioned only; (0) none mentioned.	If “AI_policy_recommend” = Yes, classify as “institutional policy recommended,” even if ethics/policy issues and instructor rules were also coded Yes. If “AI_policy_recommend” = No but “AI_policy_by_instructor” = Yes, classify as “instructor-level rules set.” If only “ethics_policy_issues_mentioned” = Yes, classify as “ethics/policy mentioned only.”

Note. SRL = self-regulated learning

## Results

### Characteristics of included studies

**Fig. 2:**
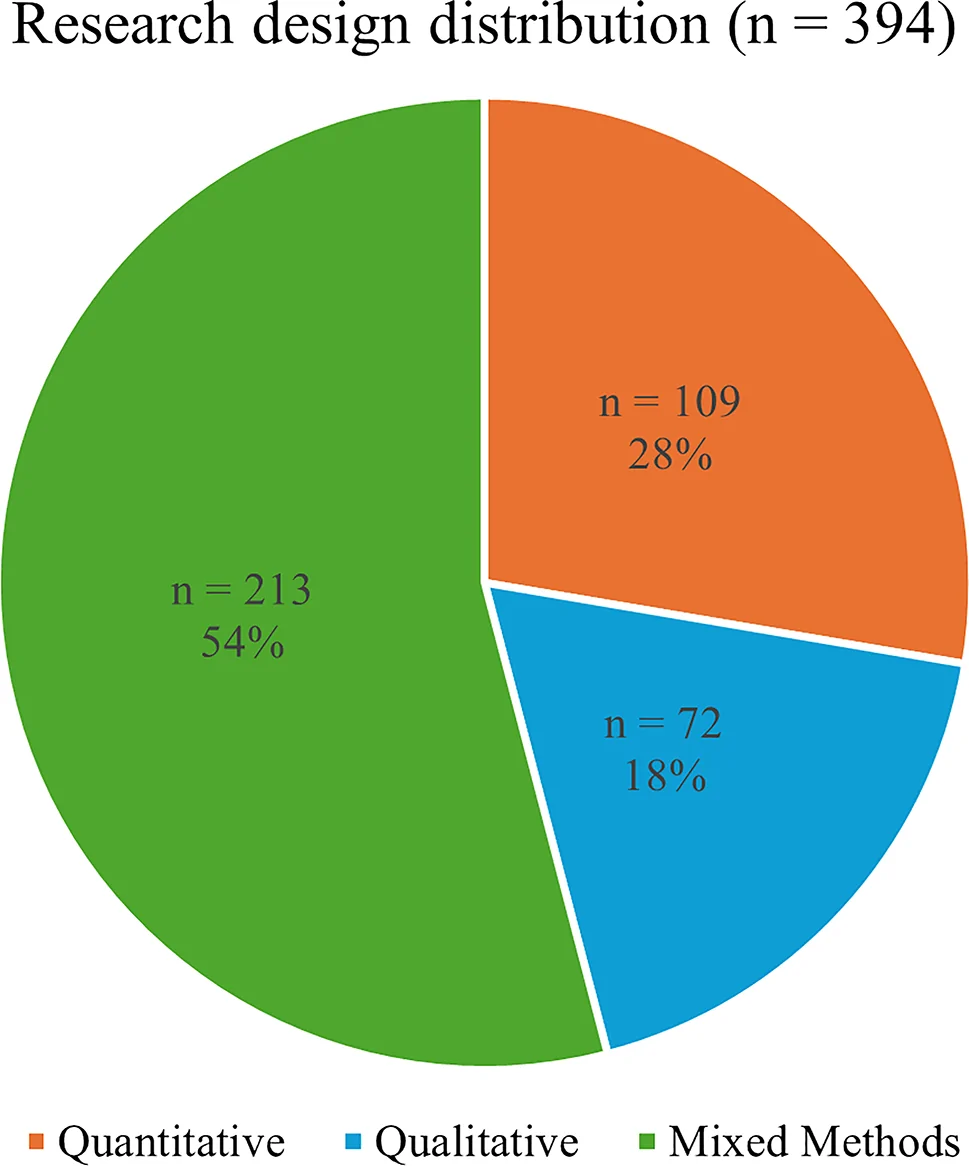
Distribution of research designs across the included studies

We first summarize the methodological and technological profile of the 394 included studies. As shown in Fig. 2, mixed methods was the most common research design (*n* = 213, 54%) followed by quantitative studies (*n* = 109, 28%) and qualitative studies (*n* = 72, 18%). This pattern represented research on enacted GenAI use in higher education employed diverse empirical designs.

**Fig. 3:**
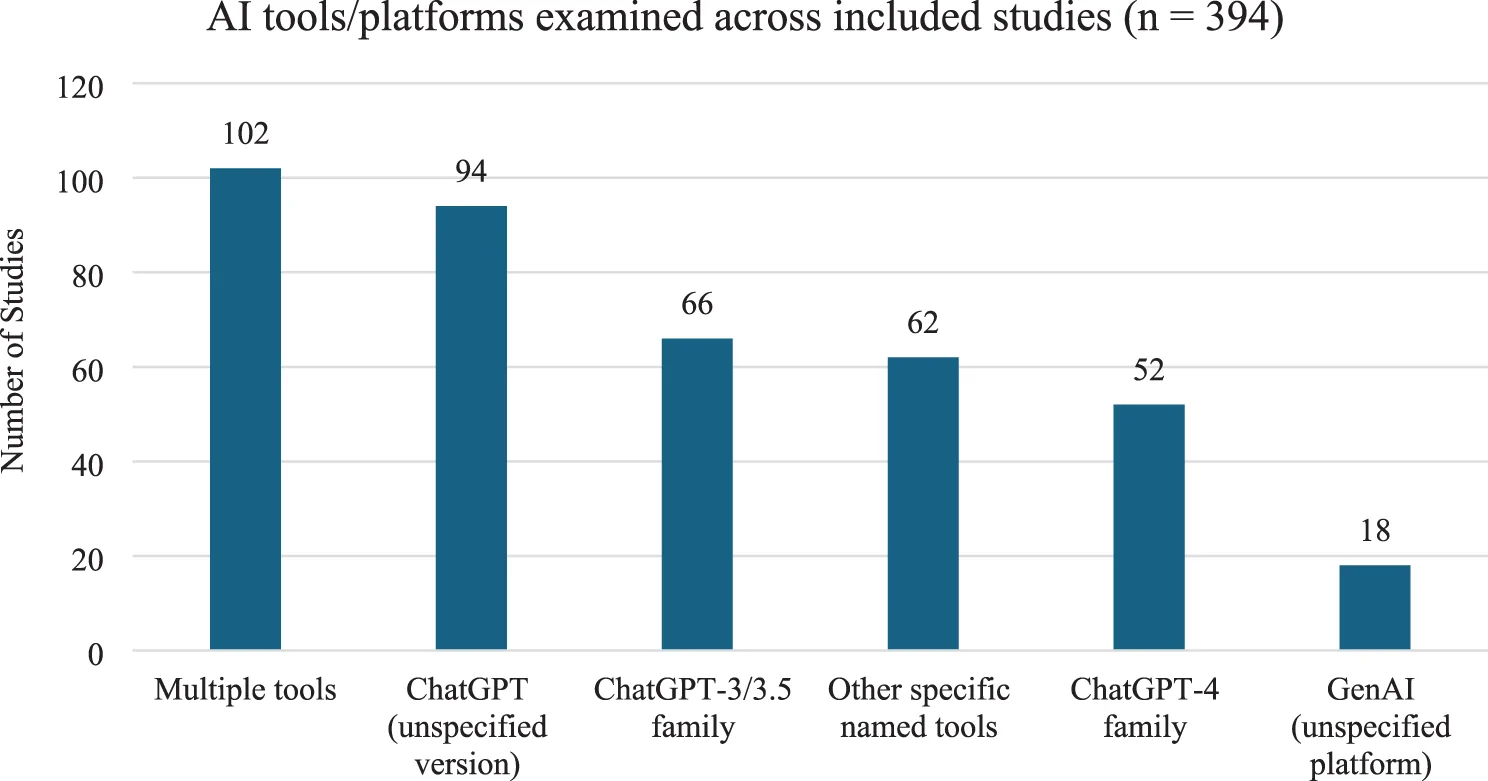
Distribution of AI tools/platforms examined across the included studies

Figure 3 presents the distribution of AI tools and platforms used across the included studies. The most common category was multiple AI tools (*n* = 102), followed by ChatGPT (unspecified version) (*n* = 94), ChatGPT-3/3.5 family tools (*n* = 66), other specific named tools (*n* = 62), and ChatGPT-4 family tools (*n* = 52). GenAI/LLM tools without a specified platform were least common (*n* = 18). As a result, ChatGPT-related applications accounted for a large share of the tool/platform categories, while other named platforms also appeared in the corpus.

**Fig. 4:**
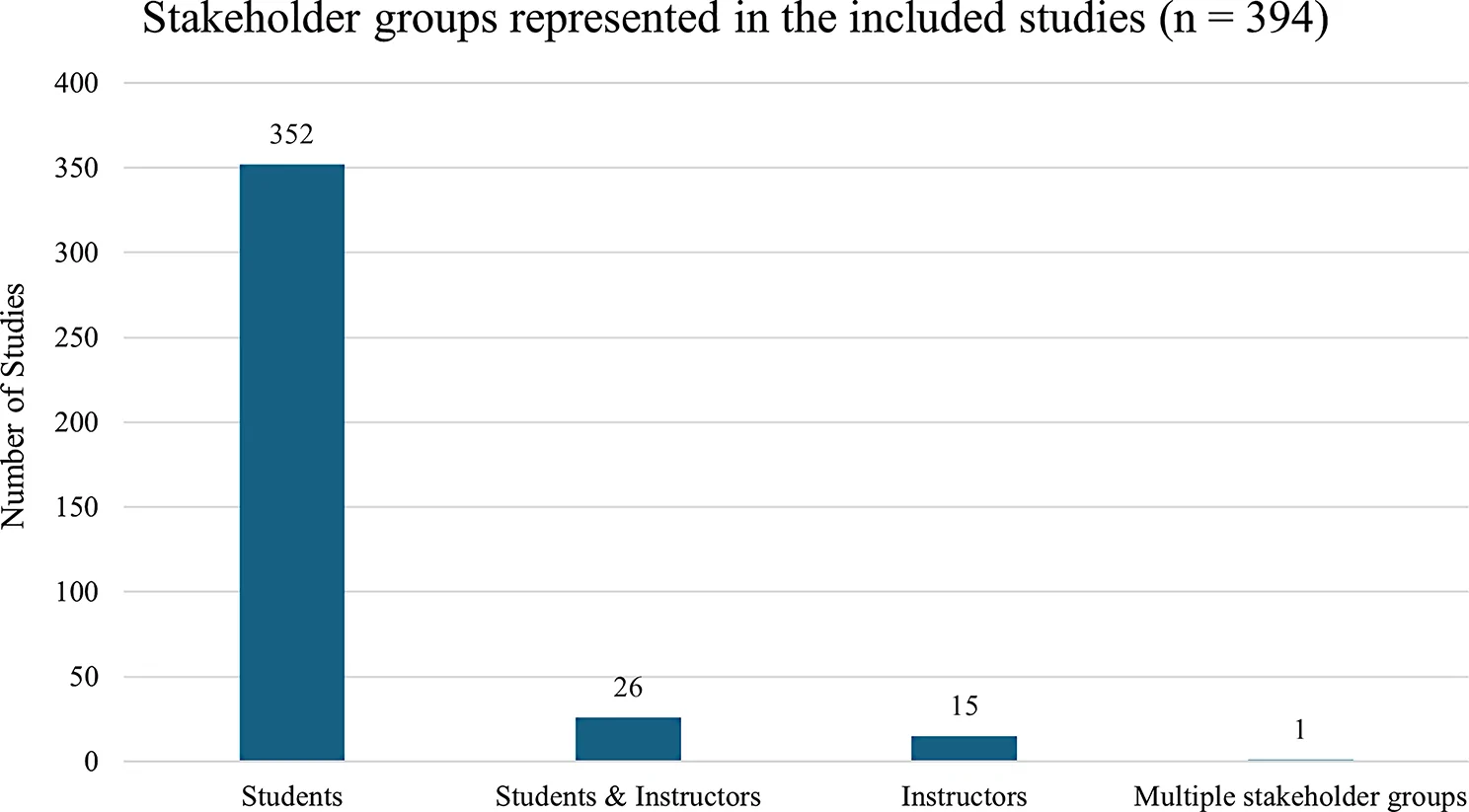
Distribution of stakeholder groups represented in the included studies

Across the 394 included studies, participant focus was coded as students (*n* = 352), instructors (*n* = 15), students and instructors (*n* = 26), and studies including multiple stakeholder groups (*n* = 1), as shown in Fig. 4. This indicates that the evidence base contributing to RQ3 was dominated by student-focused studies.

### RQ1: Use-case categories and targeted educational outcomes

**Fig. 5:**
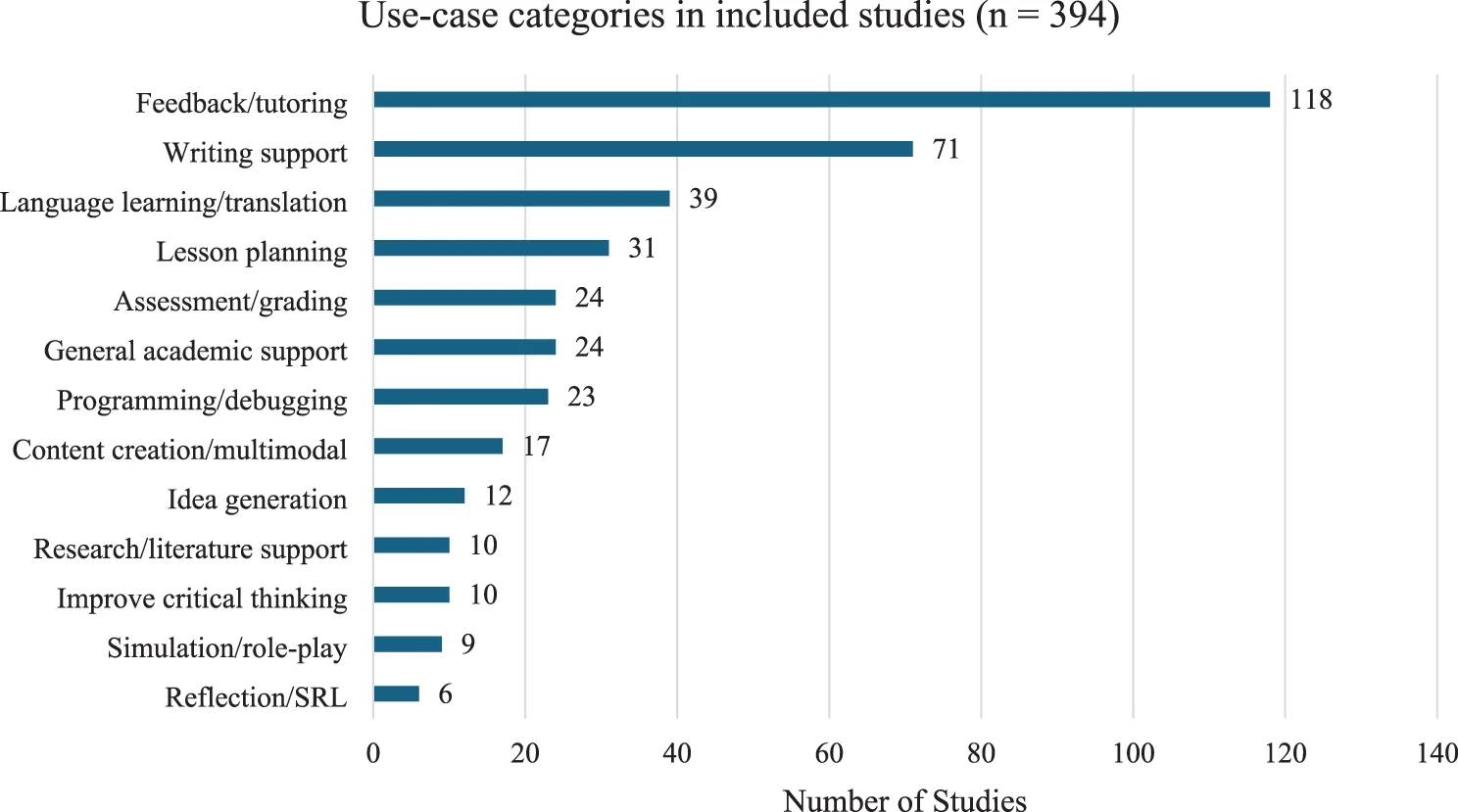
Distribution of GenAI use-case categories across the included studies

Figure 5 shows the actual use of GenAI in higher education is dominated by two instructional activities: feedback/tutoring and writing support. Other activities included lesson planning, language learning/translation, programming/debugging, and assessment/grading. Less frequent uses included idea generation, research/literature support, simulation/role-play, content creation/multimodal activities, explicit critical-thinking support, and reflection/SRL. Overall, these categories show that feedback/tutoring and writing support were the most frequently reported enacted uses in the included studies.

**Fig. 6:**
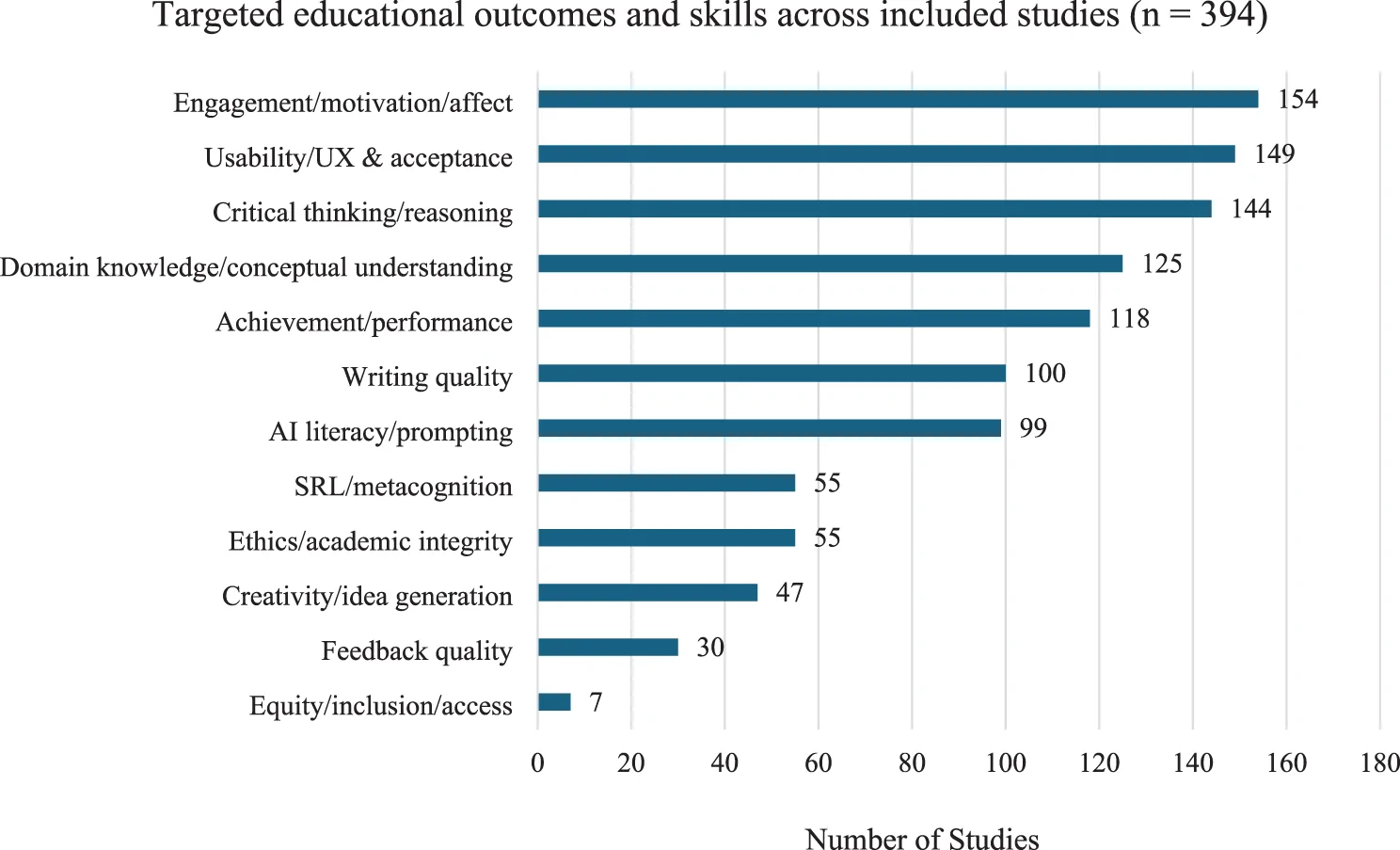
Frequency of targeted educational outcomes and skills across the included studies

Figure 6 shows the most frequently targeted outcomes were engagement/motivation/affect, usability/UX and acceptance, critical thinking/reasoning, domain knowledge/conceptual understanding, achievement/performance, writing quality, and AI literacy/prompting. Less frequently targeted outcomes included SRL/metacognition, ethics/academic integrity, creativity/idea generation, feedback quality, and equity/inclusion/access. Because studies could be coded into more than one outcome category, frequencies exceed the total number of included studies.

Table 2 shows that feedback/tutoring was the most common use case across most GenAI tool/platform categories. ChatGPT (unspecified version) and ChatGPT-3/3.5 family tools were most often associated with feedback/tutoring (*n* = 27 each), followed by writing support for ChatGPT (unspecified version) (*n* = 20) and ChatGPT-3/3.5 family tools (*n* = 10). ChatGPT-4 family tools were also most frequently linked to feedback/tutoring (*n* = 21), with smaller concentrations in writing support (*n* = 11) and assessment/grading (*n* = 7). Studies using multiple tools showed a broader distribution across use cases, including feedback/tutoring (*n* = 20), writing support (*n* = 17), lesson planning (*n* = 15), language learning/translation (*n* = 11), and assessment/grading (*n* = 7). Other specific named tools were similarly concentrated in feedback/tutoring (*n* = 21) and writing support (*n* = 11), with fewer studies addressing language learning/translation (*n* = 6) or lesson planning (*n* = 5). Overall, the cross-tabulation suggests that ChatGPT-related tools dominated feedback-oriented applications, whereas multiple-tool studies reflected somewhat broader experimentation across instructional use cases.

**Table 2:** Cross-tabulation of GenAI tool/platform categories by the six most frequent use-case categories

Tool/platform category	Feedback	Writing support	Language learning	Lesson planning	Academic support	Assessment
ChatGPT (unspecified version)	27	20	14	4	8	4
ChatGPT-4 family	21	11	3	2	1	7
GenAI/LLM unspecified platform	2	2	2	0	2	2
Multiple tools	20	17	11	15	5	7
Other specific named tools	21	11	6	5	3	1
ChatGPT-3/3.5 family	27	10	3	5	5	3

Note. Feedback = feedback/tutoring; Language learning = language learning/translation; Academic support = general academic support; Assessment = assessment/grading. Cell values represent study counts. Each study was assigned one primary tool/platform category and one primary use-case category

As shown in Table 3, feedback/tutoring showed the strongest and most distributed outcome profile across the major targeted outcomes, especially engagement/motivation/affect (*n* = 51), achievement/performance (*n* = 46), usability/UX and acceptance (*n* = 45), domain knowledge/understanding (*n* = 45), and critical thinking/reasoning (*n* = 37). Writing support was most associated with writing quality (*n* = 50), while also targeting engagement/motivation/affect (*n* = 20), critical thinking/reasoning (*n* = 19), and both usability/UX and acceptance and domain knowledge/understanding (*n* = 13 each). Language learning/translation was most often linked to engagement/motivation/affect (*n* = 20), writing quality (*n* = 13), and achievement/performance, domain knowledge/understanding, and usability/UX and acceptance (*n* = 12 each). Lesson planning emphasized usability/UX and acceptance (*n* = 16) and AI literacy/prompting (*n* = 15), whereas programming/debugging was most closely tied to achievement/performance (*n* = 16). Content creation/multimodal was linked most strongly to critical thinking/reasoning and creativity/idea generation (*n* = 10 each), while general academic support was associated most often with usability/UX and acceptance and AI literacy/prompting (*n* = 12 each). Together, these patterns indicate that GenAI in higher education has been studied most often as a feedback/tutoring and writing support tool, with targeted outcomes clustering around engagement, usability, critical thinking, achievement, knowledge development, writing quality, and AI literacy.

**Table 3:** Cross-tabulation of major use-case categories by major targeted outcomes and skills

Use-case	CT	Eng.	UX	Ach.	Know.	Writ.	AI lit.	SRL	Eth.	Creat.
Assess.	5	3	10	6	7	3	5	3	5	0
Content	10	5	6	6	4	0	6	1	2	10
Feedback	37	51	45	46	45	19	11	17	6	7
GAC	10	9	12	4	10	1	12	3	7	3
Language	7	20	12	12	12	13	4	6	4	1
Lesson	10	8	16	2	8	1	15	2	5	6
Programming	9	11	10	16	8	0	2	0	1	3
Writing	19	20	13	11	13	50	11	10	9	5

Note. Assess. = assessment/grading; Content = content creation/multimodal; Feedback = feedback/tutoring; GAC = general academic support; Language = language learning/translation; Lesson = lesson planning; Writing = writing support. CT = critical thinking/reasoning; Eng. = engagement/motivation/affect; UX = usability/UX and acceptance; Ach. = achievement/performance; Know. = domain knowledge/understanding; Writ. = writing quality; AI lit. = AI literacy/prompting; Eth. = ethics/academic integrity; Creat. = creativity/idea generation. Cell values represent use-case/outcome co-occurrences because studies could be coded into more than one outcome category

### RQ 2. Assessment methods used to evaluate targeted educational outcomes and skills

**Fig. 7:**
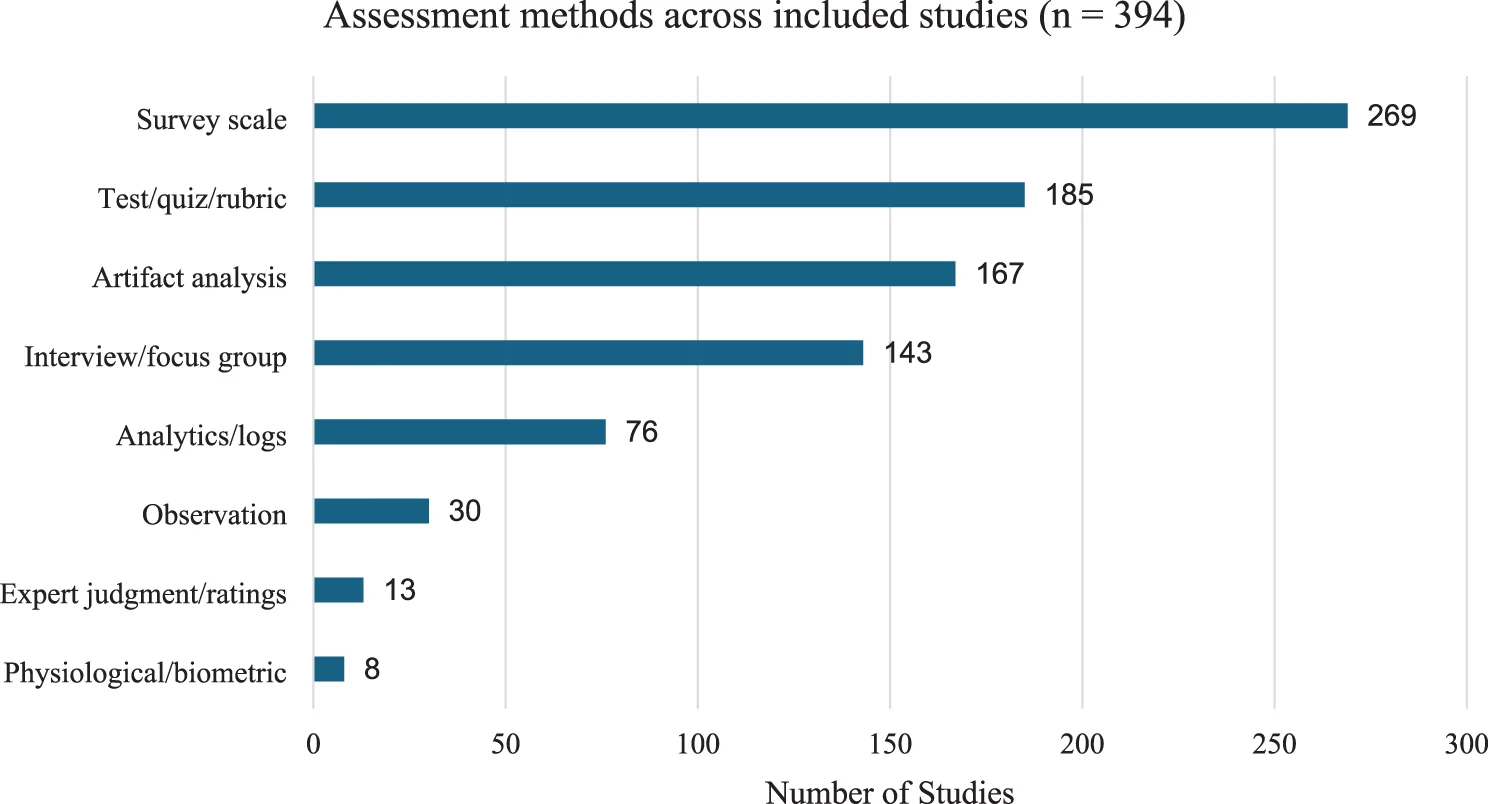
Distribution of assessment methods used across the included studies

Figure 7 presents the distribution of assessment methods used across the included studies. A total of 891 assessment-method codes were recorded across the 394 studies, reflecting the multi-label nature of this variable. Survey scales were the most frequently reported assessment method (*n* = 269; 68.3% of studies), followed by test/quiz/rubric measures (*n* = 185; 47.0%), artifact analysis (*n* = 167; 42.4%), and interview/focus group methods (*n* = 143; 36.3%). Other reported methods included analytics/logs (*n* = 76; 19.3%), observation (*n* = 30; 7.6%), expert judgment/ratings (*n* = 13; 3.3%), and physiological/biometric measures (*n* = 8; 2.0%). Because studies could employ more than one assessment method, the percentages do not sum to 100%.

### RQ 3. Student and instructor perceptions, ethics/policy signals, and governance-related reporting

Given that the included corpus was predominantly student-focused, perception evidence synthesized for RQ3 reflects student experiences more strongly than instructor perspectives. Within these enacted-use studies, perceptions were reported in relation to effectiveness, ethical concerns, and governance-related issues.

Perception direction was coded for 315 student-related cases and 35 instructor-related cases (Table 4). Among student perceptions, mixed evaluations were most common (*n* = 228, 72.4%), followed by positive evaluations (*n* = 85, 27.0%) and negative evaluations (*n* = 2, 0.6%). Among instructor perceptions, mixed evaluations were also most common (*n* = 28, 80.0%), with the remaining cases coded as positive (*n* = 7, 20.0%).

**Table 4:** Perception direction by stakeholder group

Stakeholder group	Mixed, *n* (%)	Positive, *n* (%)	Negative, *n* (%)	Total
Students	228 (72.4)	85 (27.0)	2 (0.6)	315
Instructors	28 (80.0)	7 (20.0)	0 (0.0)	35

Note. Percentages are calculated within each stakeholder group

**Fig. 8:**
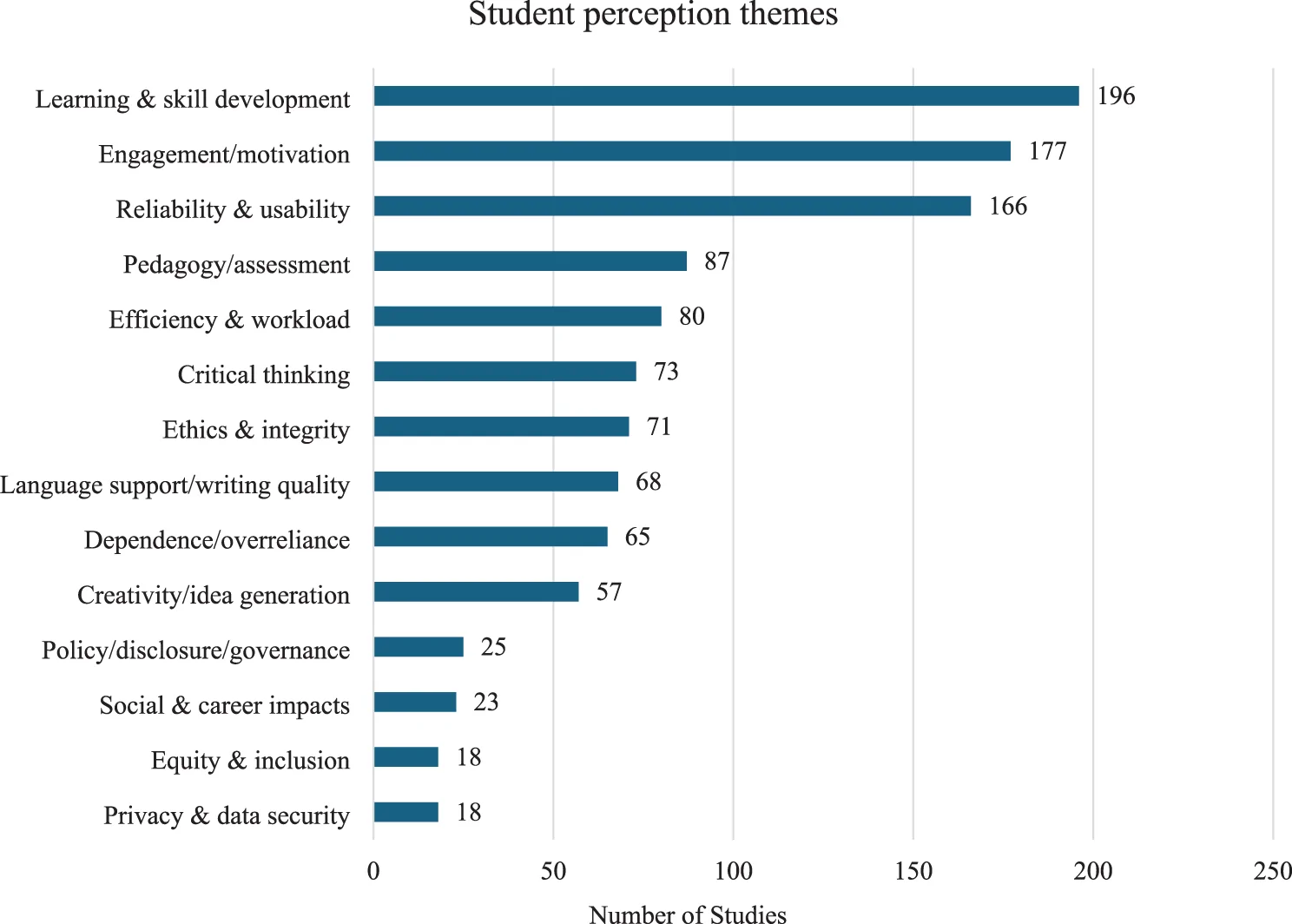
Thematic distribution of student perceptions of GenAI in higher education

Thematic coding of student perceptions is presented in Fig. 8. The most frequently coded student themes were learning and skill development (*n* = 196), engagement/motivation (*n* = 177), and reliability and usability (*n* = 166). Other commonly coded themes included pedagogy/assessment (*n* = 87), efficiency and workload (*n* = 80), critical thinking (*n* = 73), ethics and integrity (*n* = 71), and language support/writing quality (*n* = 68). Additional themes included dependence/overreliance (*n* = 65), creativity/idea generation (*n* = 57), policy/disclosure/governance (*n* = 25), social and career impacts (*n* = 23), and equity and inclusion (*n* = 18), privacy and data security (*n* = 18).

**Fig. 9:**
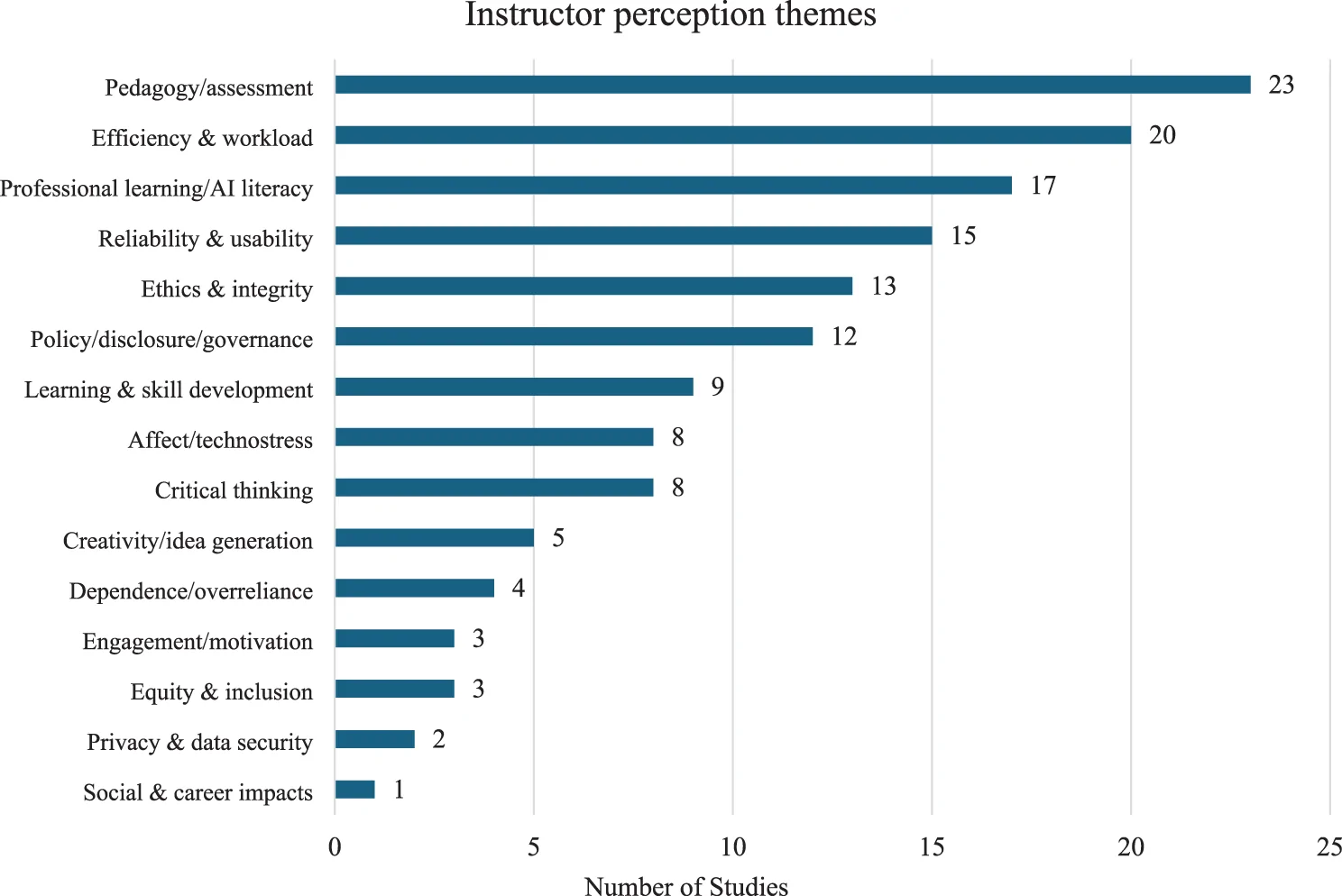
Thematic distribution of instructor perceptions of GenAI in higher education

Instructor perception themes are shown in Fig. 9. The most frequently coded instructor themes were pedagogy/assessment (*n* = 23), efficiency and workload (*n* = 20), and professional learning/AI literacy (*n* = 17). Other recurring themes included, reliability and usability (*n* = 15), ethics and integrity (*n* = 13), policy/disclosure/governance (*n* = 12), learning and skill development (*n* = 9), affect/technostress (*n* = 8), and critical thinking (*n* = 8). Less frequently coded themes included creativity/idea generation (*n* = 5), dependence/overreliance (*n* = 4), engagement/motivation (*n* = 3), equity and inclusion (*n* = 3), privacy and data security (*n* = 2), and social and career impacts (*n* = 1).

Ethics/policy and governance-related reporting was summarized using the derived policy presence variable (Table 5). Across all studies, institutional policy recommended only was the most frequent category (*n* = 151), followed by ethics/policy mentioned (*n* = 148), instructor-level rules set (*n* = 52), and none mentioned (*n* = 43). When cross-tabulated by participant focus, Institutional policy recommended was coded in instructor (*n* = 11), student (*n* = 125), and students and instructors (*n* = 15) studies. Ethics/policy mentioned only was distributed across multiple stakeholder groups (*n* = 1), instructor (*n* = 4), student (*n* = 138), and students and instructors (*n* = 5) studies. Instructor-level rules set was coded in student (*n* = 47) and students and instructors (*n* = 5) studies. None mentioned appeared in student (*n* = 42) and students and instructors (*n* = 1) studies.

**Table 5:** Policy presence by participant focus

Policy Presence	Multiple stakeholder groups	Instructors	Students	Students & Instructors	Total
Institutional policy recommended	0	11	125	15	151
Ethics/policy mentioned only	1	4	138	5	148
Instructor-level rules set	0	0	47	5	52
None mentioned	0	0	42	1	43
Total	1	15	352	26	394

Note. Policy presence is a derived four-level variable summarizing the highest level of governance explicitness reported in each study

## Discussion and conclusion

This scoping review mapped how LLM-based GenAI has been applied in higher education teaching and learning, what educational outcomes empirical studies have targeted, what assessment methods have been used to evaluate those outcomes, and how students and instructors have perceived enacted GenAI use. Across 394 studies, the field appears expansive but still consolidating. GenAI is being used across a growing range of higher education tasks, yet the evidence base remains concentrated around recurring instructional uses, readily measurable outcomes, and short-cycle assessment strategies. These findings should be interpreted as descriptive trends in the empirical literature, not as causal or effectiveness claims. As a scoping review, this study did not conduct formal critical appraisal or estimate effect sizes; accordingly, the findings indicate where empirical attention has concentrated rather than whether GenAI improves learning outcomes (Arksey & O’Malley, 2005; Levac et al., 2010; Tricco et al., 2018).

### From instructional uptake to pedagogical priorities

A central finding of this review is that GenAI use in higher education has primarily focused on a limited number of task-oriented applications. Feedback/tutoring and writing support were the most common use cases, followed by language learning/translation, lesson planning, assessment/grading, general academic support, and programming/debugging. This pattern suggests that higher education has adopted GenAI mainly as a tool for augmenting existing academic tasks, especially those involving feedback, text production, academic assistance, and instructional preparation, instead of as a catalyst for fundamentally reconfiguring curriculum or pedagogy (Bahroun et al., 2023; Jin et al., 2025). This interpretation aligns with prior AIEd and GenAI reviews showing that educational applications often emerge rapidly in practice before theoretical and pedagogical models fully explain how they support learning (Lin et al., 2026; Chen et al., 2020; Noroozi et al., 2024; Zawacki-Richter et al., 2019).

The distribution of targeted outcomes reinforces this interpretation. The most frequently coded outcomes were engagement/motivation/affect, usability/UX and acceptance, critical thinking/reasoning, domain knowledge/conceptual understanding, achievement/performance, writing quality, and AI literacy/prompting. By contrast, SRL/metacognition, ethics/academic integrity, creativity/idea generation, feedback quality, and equity/inclusion/access were less frequently emphasized. The outcome profile is revealing. On one hand, the literature is not solely focused on achievement; it also address reasoning, motivation, usability, and user experience, suggesting that researchers recognize GenAI as both a learning resource and a socio-technical intervention (Yan et al., 2024). On the other hand, the prominence of usability and acceptance indicates that much of the empirical literature remains organized around adoption-phase questions: whether GenAI tools are usable, motivating, accepted, and helpful within existing instructional arrangements.

This adoption-phase orientation is understandable in a fast-moving field, but it also creates a limitation. If research remains focused primarily on whether GenAI fits current assignments, feedback practices, and assessment routines, the field may under-examine more ambitious questions about how higher education should redesign learning activities, redistribute human and AI roles, and cultivate capacities that are not easily captured through short-term usability or satisfaction measures. Prior work has similarly argued that AIEd research needs stronger theoretical grounding and closer alignment between technology use, learning mechanisms, and educational purpose (Bauer et al., 2025; Chen et al., 2020; Noroozi et al., 2024). More ambitious studies may also require design constraints, comparison conditions, or forms of experimental control that partially interfere with naturally occurring classroom activity. This creates a methodological tension: studies need authenticity, but they also need enough structure to explain how and why GenAI may support or hinder learning.

### What counts as evidence of learning

The assessment profile identified in this review shows that empirical studies relied most often on survey scales, test/quiz/rubric measures, artifact analysis, and interview/focus group methods. These approaches are common and valuable in educational research, especially for early-stage mapping of user experience, perceived learning, and task performance. However, they constrain the strength of claims that can be made about learning processes. Self-report measures can provide important evidence about perceptions, motivation, and perceived usefulness, but they are limited indicators of learning, metacognition, or knowledge change when used alone (Brown & Zhao, 2023; Craig et al., 2020; Sitzmann et al., 2010). This is especially important because several targeted outcomes in this review, including critical thinking, feedback uptake, and SRL, are process-oriented constructs that require evidence for beyond post-task perception or single end-point measures (Barnett & Ceci, 2002; Rovers et al., 2019; Sitzmann et al., 2010; Winne, 2022).

The evidence base does extend beyond perception. Performance measures, artifact analysis, and interviews were also frequently used, suggesting that the field is beginning to examine GenAI-supported learning through multiple forms of evidence. Yet, process-focused methods such as trace data, observation, expert ratings, and physiological or biometric measures remained comparatively uncommon. As a result, much of the learning activity associated with GenAI use, including prompting, revision, feedback uptake, monitoring, verification, and reliance management, remains only partially visible (Lin et al., 2026). This limitation is consequential because GenAI use is often iterative and interactional. The current literature offers useful insight into what students and instructors report, produce, and achieve, but less into how students regulate AI use over time, how reliance develops or is restrained, and how instructional design shapes these trajectories. This gap is particularly notable given calls for more methodologically robust research on GenAI assessment and learning outcomes in higher education (Noroozi et al., 2024; Xia et al., 2024; Weng et al., 2024).

### Ambivalent uptake: students, instructors, and governance

The findings for RQ3 suggest that enacted GenAI use in higher education is characterized less by simple enthusiasm or rejection than by ambivalence, negotiation, and governance. For both students and instructors, mixed perceptions were more common than purely positive or negative perceptions. This pattern challenges polarized narratives that cast GenAI either as an unequivocal pedagogical benefit or as a straightforward threat to academic integrity. Instead, the evidence suggests that both groups are navigating its affordances and risks simultaneously, consistent with emerging work on GenAI perceptions, academic integrity, and responsible use in higher education (Colbran et al., 2026; Dai & Chan, 2026; Gruenhagen et al., 2024; Kim et al., 2025; Yusuf et al., 2024).

At the same time, students and instructors appeared to frame those tensions differently. Students most often emphasized learning and skill development, engagement/motivation, and reliability/usability, suggesting that they encounter GenAI primarily through its immediate utility for academic support, task completion, perceived learning assistance, and efficiency. Instructors, by contrast, most often emphasized pedagogy/assessment, efficiency/workload, professional learning/AI literacy, reliability/usability, and ethics/integrity. These differences point to a meaningful divergence in stakeholder standpoint. Students often approach GenAI through immediate academic tasks and outcomes, whereas instructors are more likely to evaluate GenAI in relation to longer-term learning, assessment validity, academic integrity, and professional responsibility. Prior studies of educational technology adoption similarly show that students and instructors can evaluate the same technology through different experiential and institutional lenses (Chugh et al., 2023; Li, 2007; Nikolic et al., 2024).

Both perspectives are legitimate, but they can generate tension. Students’ interest in utility and efficiency may make them less receptive to desirable difficulties that support deeper learning, while instructors’ caution about integrity and assessment validity may protect educational quality but also discourage productive experimentation (Dai & Chan, 2026; Gruenhagen et al., 2024; Kim et al., 2025). This tension is especially important in GenAI contexts because the same tool can support learning, accelerate task completion, obscure authorship, or reduce engagement with difficult cognitive work depending on task design and governance. The challenge for higher education is therefore not to privilege one perspective over the other, but to design learning environments where efficiency, experimentation, accountability, and meaningful learning are held together.

The governance findings reinforce this point. Across the reviewed studies, policy was most often framed as a general ethical or policy concern or as a recommendation for institutional policy, while fewer studies reported explicit instructor-level rules governing GenAI use in enacted learning contexts. This suggests that ethical awareness has become widespread, but concrete governance remains uneven. The field appears to be moving from recognizing the problem toward experimenting with responses. This pattern aligns with broader higher education literature calling for clearer institutional guidance, disclosure norms, and pedagogically informed policy frameworks (Chan, 2023; Ifenthaler et al., 2024; Jin et al., 2025; Wang et al., 2024; Chang et al., 2026).

Importantly, governance should not be understood only as a compliance issue. In course settings, governance includes explicit guidance about what forms of AI assistance are permitted, how students should disclose use, how AI-supported work will be assessed, and which parts of the learning process require human judgment. Without such guidance, students may interpret GenAI use primarily through convenience and task completion, while instructors may experience it primarily as a threat to assessment validity. More productive governance would support bounded experimentation, where students can learn to use GenAI responsibly while instructors retain clarity about learning goals, evidence of student understanding, and acceptable forms of support.

### Equity as a peripheral but consequential gap

The equity and inclusion findings deserve particular attention. Equity/inclusion/access was rarely targeted as an outcome, and equity-related perception themes appeared much less often than themes related to engagement, learning support, usability, pedagogy, efficiency, or integrity. This does not mean equity is unimportant in GenAI use; rather, it might suggest that equity remains peripheral in enacted higher education studies. This is notable because GenAI use may intersect with access to paid tools, linguistic privilege, disability support, uneven AI literacy, institutional rules, and discipline-specific norms (An et al., 2025; Lin, et al., 2026; Weng & Fu, 2025). These concerns are consistent with broader discussions of AI literacy, inclusion, and differential access in educational technology research (Ballantyne et al., 2025; Lin et al., 2024; Sperling et al., 2024).

The equity implications of GenAI will likely vary across jurisdictions, institutions, disciplines, and student populations, so broad claims should be made cautiously. Nevertheless, the low visibility of equity in this corpus is itself a meaningful finding. In contrast to emerging work on AI literacy and AI ethics in K-12 and teacher education contexts, higher education studies of enacted GenAI use appear to have focused more strongly on adoption, usability, performance, and integrity than on differential access or inclusion (e.g., Ma et al., 2025; Sperling et al., 2024; Wiese et al., 2025). Future studies should therefore treat equity not as a secondary concern but as a core dimension of educational impact.

### Implications for research

Several implications follow for future research. First, the field would benefit from shifting from documenting adoption toward explaining learning. Current studies provide substantial evidence about where and how GenAI is being used, but less about how these uses support or constrain learning. Research grounded in learning sciences frameworks, especially those related to feedback, self-regulation, and metacognitive monitoring, may help move the literature beyond descriptive mapping toward explanatory insight (Hattie & Timperley, 2007; Winne, 2022).

Second, future studies should better align targeted outcomes with assessment strategies. If a study claims to examine self-regulated learning, critical thinking, AI literacy, or disciplinary reasoning, these constructs should be measured in ways that go beyond self-reported satisfaction or perceived benefit. Stronger designs would combine performance measures, trace data, artifact analysis, observation, and qualitative process evidence to examine not only whether students report using AI successfully, but how they used it, under what instructional conditions, and with what trade-offs in learning, effort, and achievement.

Third, multi-stakeholder designs remain important. Because students and instructors often perceive GenAI differently, studies focusing on only one stakeholder group risk overlooking tensions that shape actual classroom use. Comparative designs examining the same instructional context from both student and instructor perspectives would be particularly valuable for understanding mismatches between learner motivations, assessment design, and institutional expectations. Such designs could examine, for example, how students use GenAI to expedite drafting while instructors use it to manage feedback or grading workload, and how these parallel uses alter the meaning of authorship, feedback, and assessment.

Fourth, more attention is needed to understand underrepresented outcomes. The relatively modest attention to self-regulated learning/metacognition, ethics/academic integrity, creativity, and especially equity/inclusion/access points toward important blind spots in the current literature. Given known concerns about uneven access, disclosure pressure, linguistic privilege, and variable AI literacy, future research should treat these not as peripheral issues but as core dimensions of GenAI’s educational impact.

### Implications for practice and policy

For practice, the findings suggest that GenAI should be treated as an instructional design issue, not simply a decision about adopting a tool. Because the most common applications involve feedback and writing support, instructors need to clarify how GenAI can contribute to those tasks, what forms of support are permitted, and where human judgment remains essential. Prior work has similarly emphasized the importance of explicit instructional guidance, transparency, and alignment between GenAI use and learning goals to reduce ambiguity and support responsible educational use (Chan, 2023; Chang et al., 2023; Jin et al., 2025; Wang et al., 2024).

For policy, the results suggest that a sole focus on ethics is insufficient. While many studies mentioned ethical or policy issues, fewer reported implemented instructor-level rules, and fewer still reflected explicit governance structures embedded in course activity. Institutions therefore need frameworks that go beyond broad warnings or prohibitions. More productive approaches are likely to emphasize transparency, disclosure, pedagogical alignment, and staff development while supporting instructors in designing assessments that remain meaningful in AI-infused environments (Chan, 2023; Ifenthaler et al., 2024; Jin et al., 2025).

The perception findings also imply that one-size-fits-all policy is unlikely to suffice. Students’ practical interest in efficiency and support, and instructors’ stronger emphasis on assessment, ethics, and workload, suggest that effective governance must address both user experience and instructional responsibility. Flexible, discipline-sensitive guidance may therefore be more effective than generic institutional statements, especially when GenAI use differs across writing-intensive, programming-intensive, clinical, professional, and design-based learning contexts (An et al., 2025; Ballantyne et al., 2025; Lin et al., 2024; Weng & Fu, 2025).

### Limitations and concluding remarks

Several limitations should be acknowledged. First, as a scoping review, this study mapped the characteristics and patterns within the evidence base but did not assess study quality or estimate effect sizes. Second, the review was intentionally restricted to empirical studies reporting enacted, task-anchored use in authentic higher education contexts. This decision enhanced conceptual precision, but it also excluded perception-only studies, conceptual papers, reviews, and studies primarily focused on evaluating AI-generated outputs as research artifacts. Third, coding necessarily involved abstraction from complex instructional settings, which may have simplified overlapping uses, outcomes, stakeholder groups, and governance considerations. Fourth, assessment methods and targeted outcomes were coded as separate study-level multi-label variables; therefore, this review reports the distribution of assessment methods across studies rather than assigning specific methods to specific outcomes within multi-method studies. Finally, the field is evolving rapidly, so the patterns reported here should be interpreted as a structured overview of a dynamic field rather than a final account of GenAI in higher education.

This scoping review shows that empirical research on GenAI in higher education has moved beyond speculative commentary and now documents a substantial body of classroom and course-related use. Meanwhile, the evidence base remains concentrated around a relatively narrow set of uses, especially feedback/tutoring and writing support, and a familiar set of outcomes, such as engagement, usability, critical thinking, achievement, knowledge development, and writing quality. Assessment has relied heavily on surveys, performance measures, artifact analysis, and interviews, while more process-sensitive approaches remain less common. Stakeholder perceptions were predominantly mixed, not polarized, and governance often appeared more as ethical concern or policy recommendation than as fully articulated instructional or institutional rule structures.

Overall, the field is no longer asking only whether GenAI can be used in higher education. It is increasingly confronting a more demanding question: under what pedagogical, assessment, and governance conditions does GenAI support meaningful learning without displacing the capacities higher education seeks to cultivate? Advancing this field will require more theoretically grounded outcome selection, stronger alignment between constructs and assessment, greater attention to self-regulation and equity, and clearer institutional frameworks that support responsible, instructionally coherent use. By clarifying current patterns and gaps, this review provides a foundation for more analytically rigorous and educationally consequential research on GenAI in higher education.

## Supplementary Information

Below is the link to the electronic supplementary material.

Supplementary Material 1

Supplementary Material 2

## Data Availability

No datasets were generated or analysed during the current study.
