# Supplementary material for: Mapping generative AI in higher education teaching and learning: a scoping review
Source: Int J Educ Technol High Educ. 2026 Oct 15;23(1):55. doi: 10.1186/s41239-026-00630-9 (PMC13668961; doi:10.1186/s41239-026-00630-9)
Supplement: Supplementary file 1 [file 41239_2026_630_MOESM2_ESM.docx]

**Appendix A. Full database-specific search strategies**

Searches were conducted in January 2026. Record counts reported in the PRISMA flow diagram reflect the records exported from each database at the time of the original search and reconciled through the RIS/Zotero export files. Because bibliographic databases are continuously updated and platform search settings may change over time, rerunning the same search at a later date may produce different hit counts. The review corpus and PRISMA counts are therefore based on the archived search exports used for screening.

Table: exact strings

| **Database / platform** | **Search/export date** | **Field searched** | **Exact search string** | **Limits / filters** | **Records exported** |
| --- | --- | --- | --- | --- | --- |
| EBSCOhost | Jan. 10, 2026 | Title or Abstract | XB (“generative AI” OR “generative artificial intelligence” OR “ChatGPT” OR “large language model*” OR “LLM*”) AND (“teaching” OR “learning” OR “instruction” OR “education”) AND (“higher education” OR “college” OR “university” OR “post-secondary” OR “postsecondary” OR “undergraduate”) | Peer-reviewed journal articles; English language; publication dates Nov. 2022–Dec. 2025 | 976 |
| Web of Science Core Collection | Jan. 10, 2026 | Title or Abstract | ((TI=(“generative AI” OR “generative artificial intelligence” OR “ChatGPT” OR “large language model*” OR “LLM*”) OR AB=(“generative AI” OR “generative artificial intelligence” OR “ChatGPT” OR “large language model*” OR “LLM*”)) AND (TI=(“teaching” OR “learning” OR “instruction” OR “education”) OR AB=(“teaching” OR “learning” OR “instruction” OR “education”)) AND (TI=(“higher education” OR “college” OR “university” OR “post-secondary” OR “postsecondary” OR “undergraduate”) OR AB=(“higher education” OR “college” OR “university” OR “post-secondary” OR “postsecondary” OR “undergraduate”))) | Document type: Article; English language; publication dates Nov. 2022–Dec. 2025 | 1,802 |

**Appendix B. Operational Codebook for Data Charting and Synthesis**

**Scope and purpose.** This codebook defines the study-level variables used to chart and synthesize empirical studies of GenAI use in higher education teaching and learning. Table 1 in the manuscript summarizes the main analytic categories; this appendix provides operational definitions, allowable values, coding rules, examples, and decision rules for ambiguous cases. Supplementary Table S1 contains the analytic study-level data used in the descriptive synthesis.

**Unit of coding.** The unit of coding was the included study. When a study reported multiple courses, activities, tools, or stakeholder groups, coders prioritized the activity, tool, or stakeholder evidence most central to the study aim, intervention, research question, or primary outcome. If multiple elements were equally central, coders recorded the dominant element used in the reported analysis and documented ambiguity in the extraction notes.

**Evidence rule.** Codes were assigned only when supported by the article text, tables, figures, instrument descriptions, results, or appendices. Coders did not infer categories from general discussion or author speculation unless the article explicitly linked the claim to the enacted study context. When information was absent or unclear, coders used “Not reported/unclear” rather than inferring a code.

| **Coding type** | **Use in this review** | **Examples** |
| --- | --- | --- |
| Single-label categorical | One code was assigned per study or stakeholder group. | use_case_category; research design; participant focus; GenAI tool/platform category; perception direction; policy presence |
| Multi-label categorical | A study could receive more than one code when multiple outcomes, assessment methods, or perception themes were empirically reported. | targeted outcomes; assessment methods; student/instructor perception themes |
| Binary | Codes were assigned as Yes/No/Not reported according to evidence in the article. | student/instructor perception examined; ethics/policy issues mentioned; AI policy recommend; AI policy by instructor |

# 1. Study profile and descriptive variables

These variables describe the included studies and support the descriptive profile of the corpus.

| **Variable** | **Role** | **Operational definition and coding rule** |
| --- | --- | --- |
| Number; Author; Year | Bibliographic / identification field | Record the study identifier, authoring information, and year of publication used to identify each included study in Supplementary Table S1. Additional bibliographic information may be retained in the master extraction spreadsheet for audit purposes. |
| participant_focus | Single-label analytic variable | Primary participant group represented in the empirical study. Allowable values: student; instructor; students and instructors; Multiple stakeholder groups. Code “students and instructors” when both groups are explicitly included and analyzed. Code “Multiple stakeholder groups” when the study includes additional groups beyond students/instructors, such as administrators, designers, employers, or institutional stakeholders. |
| research_design | Single-label analytic variable | Broad methodological design. Allowable values: Qualitative; Quantitative; Mixed methods. Code based on the article’s stated design, methods, data sources, and analysis. |
| GenAI tool/platform category | Single-label analytic variable | Primary GenAI tool category used in the study. Allowable values: Multiple tools; ChatGPT (unspecified version); ChatGPT-3/3.5 family; ChatGPT-4 family; Other specific named tools; GenAI/LLM unspecified platform. Code “Multiple tools” when more than one GenAI system was central to the study. Code “Other specific named tools” when a named tool outside the ChatGPT families was used (e.g., Gemini/Bard, Claude, DALL-E, ERNIE/Baidu-family systems, Doubao, Kimi/Moonshot, or custom GPT-based systems). Version-specific decision rule: Code “ChatGPT-3/3.5 family” when the article explicitly identifies the tool as GPT-3, GPT-3.5, ChatGPT-3.5, or free ChatGPT powered by GPT-3.5. Code “ChatGPT-4 family” when the article explicitly identifies GPT-4, ChatGPT-4, GPT-4o, or another GPT-4-family model. Code “ChatGPT (unspecified version)” only when the article names ChatGPT but does not report the model family or version. |

# 2. RQ1 variables: GenAI use cases and targeted outcomes

## 2.1 Coding inputs and use-case category assignment

Coding inputs. During data charting, coders recorded each article’s stated purpose for GenAI use and the enacted learning activity or task in the master extraction spreadsheet. In the spreadsheet, these descriptive coding inputs were recorded as Purpose of use and Learning activity/task type. They served as evidence for assigning the single-label analytic variable use_case_category and were not analyzed or reported as separate final categorical outcomes.

Analytic variable. use_case_category captured the primary enacted GenAI-supported teaching or learning activity in each study. Initial coding was informed by prior AI/GenAI education scoping review frameworks, and final categories were refined against the included corpus through iterative coding and consensus discussion. Each study was assigned only one primary use-case category based on the article’s description of the study aim, intervention, task design, enacted GenAI activity, and/or primary outcome.

| **use_case_category code** | **Operational definition** | **Decision rule / example** |
| --- | --- | --- |
| Feedback/tutoring | GenAI was used to provide formative feedback, tutoring, Q&A, hints, explanations, coaching, or individualized academic support during a learning task. | Use when feedback or tutoring was the central activity, even if the task involved writing or domain learning. Example: students received AI-generated formative comments on draft essays. |
| Writing support | GenAI was used primarily for writing, drafting, revision, grammar, style, organization, argumentation, or written communication. | Use when the central activity was producing or improving written work. If the study centered on feedback provision rather than writing production, code Feedback/tutoring. |
| Lesson planning | GenAI was used by instructors or pre-service teachers to design lessons, generate teaching materials, plan activities, or support instructional preparation. | Example: instructors used ChatGPT to generate lesson plans or classroom activities. |
| Language learning/translation | GenAI was used for language acquisition, translation, vocabulary, grammar, speaking/listening, EFL/ESL learning, or multilingual communication. | Use when the central educational task was language learning or translation rather than general academic writing. |
| Programming/debugging | GenAI was used for coding, debugging, software development, programming explanations, computational problem solving, or code review. | Example: students used ChatGPT to debug Python code or receive programming explanations. |
| Assessment/grading | GenAI was used to design, generate, score, grade, evaluate, or support assessments, rubrics, test items, feedback on assessment tasks, or grading workflows. | Use when assessment or grading was the primary activity, not merely an outcome measure. |
| Idea generation | GenAI was used primarily for brainstorming, ideation, topic generation, planning, or exploring possible approaches. | Use when idea generation was the main activity and not subordinate to writing, research/literature support, or lesson planning. |
| Research/literature support | GenAI was used to support literature search, article summarization, research design, academic source synthesis, literature review tasks, or research workflow. | Use when the activity was explicitly research- or literature-focused. |
| General academic support | GenAI was used for broad coursework help, academic assistance, studying, explanations, or task support that did not clearly fit another category. | Use as a conservative category when the enacted activity was authentic and course-related but too broad or insufficiently specific for another use-case code. |
| Simulation/role-play | GenAI was used as a simulated interlocutor, patient, client, peer, interviewee, debate partner, or role-play agent. | Example: students practiced counseling, clinical interviewing, language conversation, or negotiation with a GenAI role-play agent. |
| Content creation/multimodal | GenAI was used to create multimodal or non-standard instructional/learning artifacts such as images, videos, slides, visual designs, multimodal content, or media products. | Use when the content creation product, rather than writing alone, was central to the activity. |
| Improve critical thinking | GenAI was used explicitly to foster, challenge, scaffold, or evaluate critical thinking, reasoning, argument evaluation, or analytical judgment as the central learning activity. | Use only when critical thinking was the primary enacted activity, not merely one targeted outcome attached to another use case. |
| Reflection/SRL | GenAI was used to support reflection, SRL, learning planning, monitoring, metacognitive prompts, goal setting, or reflective journaling. | Use when reflective or self-regulatory activity was central to the intervention or task. |

**Ambiguous-case priority rule.** If a study included multiple GenAI activities, coders assigned the category most central to the research question, intervention, task design, or primary outcome. If activities were equally central, coders prioritized the activity linked to the most direct outcome evidence. Ambiguous cases were documented and resolved through consensus adjudication.

## 2.2 Targeted skills / educational outcomes

Coding type. Multi-label. Code all outcomes or skills that the study explicitly aimed to develop, measure, or evaluate in relation to GenAI use. Do not code outcomes mentioned only in the literature review, author speculation, or recommendations unless they were part of the empirical study aim, design, measures, findings, or analysis.

| **Outcome code** | **Operational definition** | **Examples / decision rules** |
| --- | --- | --- |
| critical thinking/reasoning | Reasoning, argumentation, evaluation, problem solving, analytical judgment, skepticism toward AI outputs, or higher-order thinking. | Code when the study targeted or measured critical thinking, reasoning quality, argument quality, problem-solving reasoning, or evaluation of AI-generated content. |
| engagement/motivation/affect | Learner engagement, motivation, interest, enjoyment, confidence, anxiety, attitudes, affective response, or willingness to use GenAI. | Code when affective or motivational outcomes were empirically measured or reported as outcomes. |
| usability/UX & acceptance | Usability, perceived usefulness, ease of use, user experience, technology acceptance, satisfaction, trust, or intention to use. | Code when the study assessed acceptance or interaction quality with the GenAI tool. |
| achievement/performance | Demonstrated performance on assessed tasks, such as grades, scores, rubric-based outcomes, task completion, task accuracy, or performance improvement. | Distinguish from knowledge. Code achievement when the outcome is assessed task performance or score-based evaluation. |
| domain knowledge/understanding | Subject-matter understanding, conceptual knowledge, content knowledge, or knowledge acquisition not necessarily measured through formal task performance. | Use when the article emphasizes understanding of disciplinary content or conceptual learning, rather than assessed task scores. |
| writing quality | Quality of written products, including organization, grammar, coherence, argumentation, lexical/syntactic quality, revision quality, or academic writing performance. | Code in addition to achievement if writing scores or rubrics are used. |
| AI literacy/prompting | Knowledge, skills, or dispositions related to understanding GenAI, evaluating AI outputs, prompting, responsible AI use, or AI/digital literacy. | Code when prompting skill, AI literacy, or responsible AI competence is targeted or measured. |
| SRL/metacognition | SRL related elements, such as planning, monitoring, reflection, goal setting, feedback uptake, metacognitive awareness, or learning strategy use. | Code when students’ regulation of learning or AI use is targeted or assessed. |
| ethics/academic integrity | Academic integrity, plagiarism, authorship, responsible use, bias, fairness, accountability, or ethical decision making related to GenAI. | Code as targeted outcome when ethics/integrity is taught, measured, or assessed, not merely mentioned as a concern. |
| creativity/idea generation | Creativity, originality, ideation, divergent thinking, creative confidence, or production of novel ideas/artifacts. | Code when creativity or ideation is an outcome, even if the use case is writing or content creation. |
| feedback quality | Quality, usefulness, specificity, timeliness, accuracy, or uptake of feedback generated by GenAI or mediated through GenAI. | Code when feedback itself is evaluated as an outcome. |
| equity/inclusion/access | Equitable access, inclusion, accessibility, linguistic privilege, disability support, affordability, or participation gaps. | Code when equity, inclusion, or access is empirically targeted, measured, or analyzed. |

# 3. RQ2 variables: Assessment methods

**Coding type.** Multi-label. Assessment methods were coded at the study level. Coders recorded every method used to evaluate targeted outcomes, skills, perceptions, or processes associated with GenAI use. Methods used only for background demographics or general study description were not coded unless they were part of outcome, skill, perception, or process evaluation. When a study reported multiple assessment methods and multiple targeted outcomes, coders recorded all methods and all outcomes supported by the article evidence, but did not infer method–outcome pairings. Accordingly, the synthesis reports assessment-method frequencies across studies rather than method-by-outcome pairings.

**Decision rule for compound instruments.** When a study used a questionnaire, worksheet, task sheet, or other compound instrument containing multiple components, coders assigned assessment-method codes according to the function of each component and only when that component was actually used in outcome or perception evaluation. Evidence should normally appear in the Methods, Measures, Procedure, Data collection, Data analysis, Results, tables, appendices, or supplementary materials. Methods mentioned only in the literature review, background, general discussion, or recommendations were not coded unless the article explicitly showed that they were used in the empirical study. Structured survey or questionnaire components, including closed-ended and open-ended survey items, were coded as “Survey scale” when they contributed to outcome or perception evaluation. Disciplinary questions, exams, or scored task items were coded as “Test/quiz/rubric” when used to evaluate learning or performance. Interviews and focus groups were coded as “Interview/focus group” only when the study actually conducted interviews or focus groups. Student answer scripts, assignments, AI-generated submissions, prompts, conversation records, or other submitted products were coded as “Artifact analysis” when the artifact itself was scored, compared, checked for similarity, coded, or otherwise analyzed.

| **Assessment method code** | **Operational definition** | **Examples / decision rules** |
| --- | --- | --- |
| survey scale | Closed-ended survey, questionnaire, Likert-type scale, standardized self-report instrument, or structured rating scale completed by participants. | Use for perceived usefulness, motivation, attitude, engagement, satisfaction, self-efficacy, or similar scales. |
| test/quiz/rubric | Tests, quizzes, exams, grades, pre/post assessments, rubric-based scoring, task scores, or accuracy measures used to evaluate learning or performance. | Includes instructor scoring or rubric-based evaluation of student work. |
| artifact analysis | Analysis of student products or GenAI-related artifacts, including essays, code, prompts, conversation logs, revisions, projects, or multimodal products. | Use when the artifact itself is analyzed qualitatively or quantitatively, regardless of whether a rubric is also used. |
| interview/focus group | Interviews or focus groups. | Use for any type of interviews or focus groups. |
| analytics/logs | Learning analytics, platform logs, prompt logs, interaction traces, clickstreams, revision histories, or usage data. | Use when trace data or logs are analyzed as evidence of behavior, process, or engagement. |
| observation | Classroom observation, researcher observation, field notes, video observation, or behavioral observation during the activity. | Use when observed behavior forms part of the evidence base. |
| expert judgment/ratings | Ratings by external experts, evaluators, judges, or panels, especially when distinct from course grades or instructor rubrics. | Use for expert evaluation of artifacts, AI outputs, or student performance. If the course instructor used a standard rubric, code Test/quiz/rubric. |
| physiological/biometric | Physiological, biometric, eye-tracking, EEG, sensor, or emotion-recognition data used as evidence. | Use only when such data were collected and analyzed in the empirical study. |

# 4. RQ3 variables: Student and instructor perceptions

**Evidence rule. Codes were assigned only when supported by the article text, tables, figures, instrument descriptions, results, or appendices. Coders did not infer categories from general discussion or author speculation unless the article explicitly linked the claim to the enacted study context. When information was absent or unclear, coders used “Not reported/unclear.”**

## 4.1 Perception direction

| **Direction code** | **Operational definition** | **Decision rule / example** |
| --- | --- | --- |
| Positive | Perceptions were predominantly favorable toward GenAI use, with benefits emphasized and no substantive concerns reported for that stakeholder group. | Example: students reported that GenAI was useful, convenient, and supportive, with no meaningful negative or ambivalent evidence reported. |
| Negative | Perceptions were predominantly unfavorable, with risks, harms, rejection, or concerns outweighing reported benefits for that stakeholder group. | Example: instructors described GenAI as undermining learning or assessment validity without reporting meaningful perceived benefits. |
| Mixed | Both benefits and concerns were substantively reported, or the study reported ambivalence, conditional acceptance, subgroup differences, or tradeoffs. | Code Mixed when stakeholders valued efficiency or learning support but also raised concerns about accuracy, integrity, dependence, workload, or governance. |
| Not reported | The article did not report empirical perceptions for the stakeholder group. | Use separately for students and instructors when that group was not included or perceptions were not reported. |

## 4.2 Perception themes

**Coding type.** Multi-label. Code all empirically reported themes for each stakeholder group. A single study could receive multiple perception theme codes for students and/or instructors.

| **Perception theme code** | **Operational definition** | **Student allowed?** | **Instructor allowed?** | **Examples / stakeholder notes** |
| --- | --- | --- | --- | --- |
| engagement/motivation | Interest, engagement, motivation, enjoyment, attention, confidence, willingness to participate, or affective investment in learning with GenAI. | Yes | Yes | Students reported GenAI increased motivation or made tasks more engaging. |
| learning & skill development | Perceived improvement in learning, understanding, skill development, or academic support. | Yes | Yes | Students or instructors perceived GenAI as supporting learning or skill development. |
| reliability & usability | Ease of use, usefulness, convenience, accuracy, reliability, trustworthiness, hallucination concerns, or technical usability. | Yes | Yes | Includes positive usability, concerns about inaccurate, or unreliable outputs. |
| pedagogy/assessment | Implications for teaching, learning design, assessment design, validity, feedback practice, grading, or instructional alignment. | Yes | Yes | Common for instructor perceptions about assessment redesign or validity. |
| efficiency & workload | Time savings, productivity, workload reduction, workload increase, task acceleration, or administrative efficiency. | Yes | Yes | Students may foreground efficiency; instructors may discuss workload implications. |
| language support/writing quality | Support for language learning, translation, grammar, fluency, writing quality, revision, or academic communication. | Yes | No | Especially relevant to writing and language-learning studies. |
| critical thinking | Perceived effects on judgment, reasoning, problem-solving, analytical thinking, evaluation of AI outputs, cognitive effort, skepticism, independent thinking, or higher-order thinking. | Yes | Yes | Code perceptions that GenAI improves or weakens judgment, reasoning, problem-solving, evaluation of AI outputs, or independent thinking. |
| ethics & integrity | Academic integrity, plagiarism, cheating, authorship, disclosure, responsible use, fairness, bias, or ethical concerns. | Yes | Yes | Do not confuse with the binary ethics/policy indicator; this code captures stakeholder perceptions. |
| creativity/idea generation | Perceived effects on creativity, brainstorming, originality, ideation, or creative confidence. | Yes | Yes | Code positive creativity support and concerns about homogenization or reduced originality. |
| dependence/overreliance | Concerns about dependence, automation bias, reduced effort, loss of skills, outsourcing learning, or uncritical reliance on GenAI. | Yes | Yes | Code mentions of dependence, overreliance, reduced independent capability, skill erosion, outsourcing learning or writing to GenAI, or relying on AI outputs without sufficient human effort or judgment. |
| social & career impacts | Perceived effects on employability, professional readiness, workplace expectations, peer interaction, social learning, or future careers. | Yes | Yes | Code when participants link GenAI to social participation or career preparation. |
| equity & inclusion | Access, affordability, linguistic privilege, disability support, inclusion, differential AI literacy, or uneven benefits/risks across groups. | Yes | Yes | Code when stakeholders discuss access or inclusion implications. |
| privacy & data security | Privacy, data protection, surveillance, consent, confidentiality, or data security concerns. | Yes | Yes | Code participant-reported or instructor-reported privacy concerns. |
| policy/disclosure/governance | Need for rules, disclosure, acceptable-use guidance, institutional policy, course-level policy, or governance clarity. | Yes | Yes | Use for stakeholder perceptions about policy; policy presence is coded separately from article-level evidence. |
| professional learning/AI literacy | Instructor professional development, AI literacy needs, pedagogical training, assessment literacy, or capacity-building for responsible GenAI use. | No | Yes | Primarily used for instructor perceptions but may be applied to students when AI literacy needs are perceived. |
| affect/technostress | Anxiety, stress, uncertainty, fear, excitement, overwhelm, resistance, or technostress related to GenAI adoption. | No | Yes | Commonly used for instructor affective responses but can apply to students when reported. |

Codes were applied separately to student_perception_themes and instructor_perception_themes using stakeholder-specific allowable values. When a theme was empirically present but not included in the allowable values for that stakeholder group, it was recorded in extraction notes but not entered into the formal analytic theme variable.

# 5. Ethics, policy, and governance variables

| **Variable** | **Coding type** | **Operational definition and rules** |
| --- | --- | --- |
| ethics_policy_issues_mentioned | Binary: Yes/No | Code Yes when the study addressed ethical considerations, policy implications, governance, responsible use, academic integrity, bias, privacy, accountability, disclosure, or AI-use guidelines in relation to the enacted educational context. Mentions may appear in findings, discussion, implications, or study context. |
| AI_policy_recommend | Binary: Yes/No | Code Yes when authors (not the course instructor) explicitly recommended a concrete policy, guideline, rule set, governance action, or institutional/departmental/program/course-level practice in their own voice, typically in Discussion, Conclusion, or Implications. General statements that ethics is important were insufficient unless a concrete action was recommended. |
| AI_policy_by_instructor | Binary: Yes/No | Code Yes when the study reported explicit instructor/course rules or parameters governing GenAI use in the learning task, such as allowed/prohibited uses, disclosure requirements, prompting boundaries, citation rules, or structured acceptable-use frameworks. Do not code Yes when GenAI use was merely part of the intervention procedure, tool access, training workshop, or assigned learning activity, unless the article reports explicit boundaries, conditions, or governance parameters for that use, such as allowed/prohibited uses, disclosure or citation requirements, prompting constraints, verification requirements, source-use rules, or a structured acceptable-use framework. |
| policy_presence | Derived four-level single-label variable | Derived after coding the three binary ethics/policy indicators. “policy_presence” represents the highest level of governance explicitness reported in a study and is used only as a single-label summary variable for descriptive synthesis. The three underlying binary indicators may co-occur, but “policy_presence” assigns each study to only one category using the hierarchy: policy recommendation > instructor/course rules > ethics/policy mention only > none. |

**Policy presence derivation.** The three binary indicators were first coded separately. The derived “policy_presence” variable was then generated using the following hierarchy: if “AI_policy_recommend” = Yes, classify as “institutional policy recommended,” regardless of the other two indicators. Else, if “AI_policy_by_instructor” = Yes, classify as “instructor-level rules set.” Else, if “ethics_policy_issues_mentioned” = Yes, classify as “ethics/policy mentioned only.” Else, classify as “none mentioned.” Thus, when all three binary indicators were coded Yes, the study was assigned to “institutional policy recommended” for the derived summary variable, while the underlying binary codes remained preserved in the dataset.

# 6. Calibration and adjudication

Before full data charting, three researchers jointly coded an initial calibration set of 10 included studies using the operational codebook. The purpose of this calibration step was to align interpretations of the eligibility criteria, coding categories, evidence rules, and decision rules for ambiguous cases. After calibration, the remaining included studies were divided among the three researchers for primary charting. Ambiguous cases were flagged in the master spreadsheet and discussed during team check-ins; when needed, a second researcher reread the study before a final consensus decision was entered.
